# Preparation of MS2 Phage-Like Particles and Their Use As Potential Process Control Viruses for Detection and Quantification of Enteric RNA Viruses in Different Matrices

**DOI:** 10.3389/fmicb.2016.01911

**Published:** 2016-12-01

**Authors:** Pavel Mikel, Petra Vasickova, Radek Tesarik, Hana Malenovska, Pavel Kulich, Tomas Vesely, Petr Kralik

**Affiliations:** ^1^Veterinary Research Institute, Department of Food and Feed SafetyBrno, Czechia; ^2^Department of Experimental Biology, Faculty of Science, Masaryk UniversityBrno, Czechia

**Keywords:** RT-qPCR, RNA virus, process control virus, isolation, detection, quantification, extraction efficiency calculation, MS2 phage-like particle

## Abstract

The detection and quantification of enteric RNA viruses is based on isolation of viral RNA from the sample followed by quantitative reverse transcription polymerase chain reaction (RT-qPCR). To control the whole process of analysis and in order to guarantee the validity and reliability of results, process control viruses (PCV) are used. The present article describes the process of preparation and use of such PCV– MS2 phage-like particles (MS2 PLP) – in RT-qPCR detection and quantification of enteric RNA viruses. The MS2 PLP were derived from bacteriophage MS2 carrying a unique and specific *de novo*-constructed RNA target sequence originating from the DNA of two extinct species. The amount of prepared MS2 particles was quantified using four independent methods – UV spectrophotometry, fluorimetry, transmission electron microscopy and a specifically developed duplex RT-qPCR. To evaluate the usefulness of MS2 PLP in routine diagnostics different matrices known to harbor enteric RNA viruses (swab samples, liver tissue, serum, feces, and vegetables) were artificially contaminated with specific amounts of MS2 PLP. The extraction efficiencies were calculated for each individual matrix. The prepared particles fulfill all requirements for PCV – they are very stable, non-infectious, and are genetically distinct from the target RNA viruses. Due to these properties they represent a good morphological and physiochemical model. The use of MS2 PLP as a PCV in detection and quantification of enteric RNA viruses was evaluated in different types of matrices.

## Introduction

The quantitative reverse transcription polymerase chain reaction (RT-qPCR) assay is nowadays considered as the gold standard method for detection and quantification of enteric RNA viruses such as hepatitis A virus (HAV), hepatitis E virus (HEV) or human noroviruses (NoV) (Mattison et al., 2009; Blaise-Boisseau et al., 2010; Di Pasquale et al., 2010; Vasickova et al., 2012; Hennechart-Collette et al., 2014). These enteric viruses have a significant impact on human health throughout the world. Worldwide, HAV infections account for 1.4 million cases annually and about 102 million asymptomatic and symptomatic cases occurred all together in 2013 (WHO, 2012; Vos et al., 2015), HEV infections account for 20 million cases annually (Lozano et al., 2012; Rein et al., 2012) and NoV is responsible for approximately 90% of epidemic non-bacterial outbreaks of gastroenteritis around the world (Lindesmith et al., 2003). RT-qPCR is a fast and sensitive method capable of detecting as few as 10 genome copies of viral nucleic acid in a sample (Puig et al., 2002). Because of the drawbacks of RT-qPCR including the necessity of monitoring the efficiency of concentration and RNA extraction steps, the removal of reverse transcription (RT) and PCR inhibitors there is a need of developing an entire RT-qPCR assay with a system of controls. Effective control of all the analytical steps is now required for diagnostic assays and generally involves the utilization of a non-pathogenic virus – process control virus (PCV) – which is added in a defined amount to the sample prior to the processing. Viral concentration and isolation procedures from complex matrices (e.g., food matrices or environmental samples) are often laborious and time-intensive, which increases the likelihood of mistakes that may lead to the failure of the analysis. Therefore, in 2013 the European Committee for Standardization (CEN) released ISO technical specifications (ISO/TS) ISO/TS 15216-1 and ISO/TS 15216-2 (The methods for determination of HAV and NoV in food using RT-qPCR), which require the use of PCV together with external control RNA (EAC) in RT-qPCR detection of these viruses in such complex matrices. According to these technical specifications, a cultivable non-enveloped positive-sense single stranded RNA (+ssRNA) virus shall be used as such a control. Furthermore, the PCV should be of a similar size to the target virus to provide a good morphological and physicochemical model, should be genetically distinct from the target virus to avoid cross-reactivity, and should not be naturally present in the analyzed sample.

Based on these recommendations, MS2 phage-like particles (MS2 PLP) that could be used as PCV for RT-qPCR detection of enteric RNA viruses were prepared. The technology for production of MS2 PLP is theoretically well established and uses the knowledge gained from the study of the familiar bacteriophage MS2 (*Leviviridae*, +ssRNA) (Pickett and Peabody, 1993; DuBois et al., 1997; Pasloske et al., 1998; Cheng et al., 2006; Wei B.J. et al., 2008; Yu et al., 2008). The use of wild-type bacteriophage MS2 instead of MS2 PLP has two major disadvantages. First, wild-type MS2 bacteriophage has the ability to proliferate – theoretically, in a specific samples such as those with fecal contamination that naturally contain *Escherichia coli* (*E. coli*), MS2 bacteriophage can proliferate and exceed the number of detected pathogenic RNA viruses present in the sample. Second, the wild-type MS2 bacteriophage cannot be used as a highly specific PCV because its genome does not contain the specific target sequences. In contrast with wild-type bacteriophage MS2, MS2 PLP cannot replicate and allow packaging of the specific control RNA sequence into their capsid. The advantages of MS2 PLP include their ability to protect the control RNA contained in their capsid from degradation by ubiquitous ribonucleases, their non-pathogenicity to humans, and their stability during long-term storage. The particles have similar physiochemical properties as the wild-type bacteriophage MS2, which has been used many times as a PCV in the detection of enteric RNA viruses (Dreier et al., 2006; Rolfe et al., 2007; Blaise-Boisseau et al., 2010; Shulman et al., 2012) and as the surrogate virus in enteric RNA virus environmental stability studies (Shin and Sobsey, 2003; Bae and Schwab, 2008; Park and Sobsey, 2011). The present article is a follow-up study of a previously published theoretical concept (Mikel et al., 2015) and describes a method of preparation of MS2 PLP carrying a specific control sequence and their use as a PCV in RT-qPCR detection and quantification of enteric RNA viruses in swab, liver tissue, serum, feces, and vegetable samples.

## Materials and Methods

### Construction of Control Sequence

The specific control sequence was derived from mitochondrial DNA (mtDNA) sequences of two extinct species – thylacine (*Thylacinus cynocephalus*, GenBank accession No. FJ515781.1), and the moa bird (*Dinornis struthoides*, GenBank accession No. AY326187.1). Due to the fact that the sequence was derived from mDNA of two extinct species, its natural occurrence in the analyzed samples is highly unlikely. The control sequence with a total length of 150 bp was synthesized *de novo* (Elisabeth Pharmacon, Czech Republic). The specificity of the control sequence was verified using BLAST^1^. It was also analyzed using OligoAnalyzer 3.1 software^2^ for the ability to form secondary structures, such as hairpins, which could result in unsuitability for RT or/and qPCR.

#### Preparation of Insert

The fragment encoding the control sequence derived from thylacine and the moa bird was obtained by PCR using a *de novo* template and the primer pairs TM-BlpI F and TM-HindIII R, which included *Bl*pI and *Hin*dIII restriction enzyme sites (**Table 1**). The composition of the reaction mixture was 12.5 μl of Fast start PCR master (Roche Molecular Diagnostics, Germany), 7.5 pmol of each primer and 3.3 pmol of template DNA. The assay was run in a total volume of 25 μl under the following conditions: 95°C for 3 min, followed by 25 cycles of 94 °C for 30 s, 56°C for 30 s and 72°C for 30 s and a final extension of 72°C for 5 min. PCR products were examined using agarose gel electrophoresis (1%) staining with ethidium bromide, and were purified using the QIAquick PCR purification kit (Qiagen, Germany) and subsequently digested at 37°C for 2 h. The composition of the restriction enzyme digest reaction was 500 ng of PCR product, 2 μl NEBuffer 2 (New England Biolabs, UK; NEB), 100 U *Hin*dIII and 10 U *Bl*pI endonucleases (both NEB) in a final volume of 20 μl. The cleaved PCR product was further purified using the QIAquick PCR purification kit (Qiagen).

**Table 1:** Specific oligonucleotides used in the present study.

Name	Target	Sequence
TM-BlpI F		5′-GATACAGCTCAGCCGCTTCCGTCAAACCCCTAA-3′
TM-HindIII R		5′-TAGGAAGCTTGGGTTTAGAATGTTTTCTCCCGT-3′
TM F	PCV	5′-CGCTTCCGTCAAACCCCTAA-3′
TM R	PCV	5′-GGGTTTAGAATGTTTTCTCCCGT-3′
TM P	PCV	5′-FAM-TGCTAATGTGGTGATTGCGTGTG-BHQ1-3′
NP F	IAC	5′-AGAGGACCGGGATATTCGAC-3′
NP R	IAC	5′-AGGTAGTCCGAGGAAAACTCTAAAC-3′
NP P	IAC	5′-Cy5-AGGCTCTTCTATGTTCTGACCTTGTTGGA-
		BHQ2-3′
M13 uni		5′-TGTAAAACGACGGCCAGT-3′
M13 rev		5′-CAGGAAACAGCTATGACC-3′


*Underlined sequences are *Bl*pI or *Hin*dIII restriction enzyme sites. PCV, process control virus; IAC, internal amplification control.*

#### Preparation of Vector

*Escherichia coli* BL21 (DE3) carrying the AU2 plasmid, which contains a fragment encoding MS2 maturase and coat protein was obtained from the Belgian coordinated collections of micro-organisms (BCCM, collection No. LMBP 7766). Purified plasmid AU2 (NucleoSpin Plasmid kit; Macherey-Nagel, Germany) was cleaved with *Bl*pI and *Hin*dIII restriction endonucleases at 37°C for 2 h. The composition of the restriction enzyme digest reaction was 500 ng of plasmid DNA, 5 μl NEBuffer 2, 100 U *Hin*dIII and 10 U *Bl*pI endonucleases in a final volume of 50 μl. The AU2 plasmid was dephosphorylated using 0.25 U calf intestinal alkaline phosphatase (NEB) added to the restriction mixture. The dephosphorylation reaction was run at 37°C for 1 h. Subsequently, plasmid DNA was purified using the QIAquick PCR purification kit (Qiagen).

#### Ligation of Insert and Vector, Transformation of Competent Cells and Verification of Construct

Ligation of insert and AU2 vector DNA was performed using a Quick-ligation kit (NEB) according to the manufacturer’s instructions. Transformation of *E. coli* TOP10 chemically competent cells (Life Technologies, USA) was performed according to the manufacturer’s recommendations. *E. coli* TOP10 cells were chosen for their high transformation efficiency, which provides for a large number of successfully transformed colonies. Transformed cells were grown on Luria Bertani (LB; Sigma–Aldrich, Czech Republic) agar plates containing 30 μg/ml of kanamycin (Sigma-Aldrich) overnight. Plasmid DNA was purified (NucleoSpin Plasmid kit; Macherey-Nagel) from twenty randomly selected colonies and the presence of specific inserts was confirmed by PCR using vector-specific (M13 uni and M13 rev) and insert-specific (TM F and TM R) primers (**Table 1**) as well as by sequencing (Eurofins MWG Operon, Germany).

### Transformation of *E. coli* BL21 (DE3) Cells and Induction of MS2 PLP Expression

Recombinant plasmid DNA (pAU2-TM) carrying the specific control sequence derived from thylacine and the moa bird was transformed into *E. coli* BL21 (DE3) cells (NEB) according to the manufacturer’s instructions and the bacterial cells were grown in LB broth containing 30 μg/ml of kanamycin at 37°C until the culture reached an optical density of 600 nm (OD_600_) = 1.7.

Two milliliter of the culture were transferred to 200 ml of LB broth containing 30 μg/ml of kanamycin, cultivated at 37°C until OD_600_ = 0.8 and centrifuged at 600 × *g* for 10 min at room temperature. Subsequently, the pellet was resuspended in 200 ml of LB broth containing 30 μg/ml of kanamycin. Protein expression was induced by addition of 1 mM isopropyl-L-thio-D-galactopyranoside (IPTG; Sigma–Aldrich) at 37°C for 16 h. The cell suspension was centrifuged at 600 × g for 10 min at 4°C and cells were lyzed by ultrasonic disruption (Bandelin VW3100 sonicator, probe MS73, Bandelin, Germany) at amplitude 50%, and with four pulses for a total length of 2 min at 4°C. To verify the production of MS2 coat protein sodium dodecyl sulfate-polyacrylamide gel electrophoresis (SDS-PAGE) and immunoblot analysis was carried out using a phage MS2 Coat Protein polyclonal antibody (Merck Millipore, USA) as primary antibody and Goat Anti-Rabbit IgG, Fc specific fragment (Jackson ImmunoResearch, UK) as secondary antibody.

Intact MS2 PLP were verified by transmission electron microscopy (TEM; electron microscope Philips EM 208; FEI, Czech Republic) at 18,000 × magnification and an accelerating voltage of 80 kV.

### Purification of MS2 PLP

To eliminate free nucleic acids the suspension containing lyzed cells and MS2 PLP was briefly centrifuged at 6700 × *g* for 15 min at 4°C and the supernatant (1 ml) was incubated with 100 U of DNase I (NEB) and 50 U of RNase A (Qiagen) at 37°C for 40 min. The particles were subsequently purified using sucrose density gradient ultracentrifugation. Briefly, 1 ml of supernatant was applied to the ultracentrifugation tubes, where two sucrose density layers of 25 and 45% were layered. Ultracentrifugation was conducted at 195000 × *g* (Rotor SW 55 Ti, Beckman, USA) for 3 h at 5°C. A strong source of light – LED – was used to distinguish three opalescent layers inside the ultracentrifugation tube. The three layers were checked for the presence of MS2 PLP by agarose gel electrophoresis (1%) staining with ethidium bromide. The top layer was removed by a needle and 1 ml syringe and the obtained MS2 PLP were dialyzed using a Float-A-Lyzer G2 membrane (molecular weight cut off 1000 kDa; Spectrum Laboratories, Netherlands) against 10 mM Tris containing 100 mM NaCl and 1 mM EDTA for 20 h at 4°C with three buffer changes. The purity of obtained MS2 PLP was controlled using agarose gel electrophoresis and TEM as described above.

### Quantification of MS2 PLP and Verification of Their Stability

The number of MS2 PLP was determined by UV spectrophotometry using the Avogadro constant, extinction coefficient of 0.125 mg/ml of MS2 bacteriophage per absorbance unit at 260 nm and a molecular weight of 3.0 × 10^6^ as was previously described (Cheng et al., 2006). Sample absorbance at 260 nm was measured six times and mean and standard deviation values were calculated. On the basis of these data the quantity of MS2 PLP was calculated as follows:

$$\text{Number of MS2 PLP = }\frac{x\times y}{z}\,$$

where *x* = Avogadro constant (6.023 × 10^23^/mol), *y* = extinction coefficient of MS2 bacteriophage (0.125 × 10^-3^ g/ml) and *z* = molecular weight of MS2 bacteriophage (3.0 × 10^6^ g/mol). The result of this calculation revealed that the concentration of MS2 PLP in a suspension of OD_260_ was 1 is 2.5 × 10^13^ particles/ml.

As a second method for MS2 PLP quantification TEM was used according to (Malenovska, 2013) with small modifications; the grid was stained with 2% ammonium molybdate (pH = 7.0) for 2 min, the latex standard (Agar Scientific, Stansted, England) had a concentration of 1.2 × 10^12^ particles/ml and the used magnification was 28,000 × to 36,000 × (Malenovska, 2013). MS2 PLP was counted in 300 randomly selected fields of view.

A third method for MS2 PLP was quantification by fluorimetric measurement of protein concentration using the Qubit Protein Assay Kit (Life Technologies) and Qubit 3.0 fluorometer (Life Technologies). The suspension containing MS2 PLP was diluted 10-fold in water and fluorimetric calculation of protein concentration in the samples was done according to manufacturer instructions. The samples were analyzed in hexaplicates and the mean and standard deviation values were calculated. As a fourth method for MS2 PLP quantification RT-qPCR was used as described below.

Stability of MS2 PLP was verified by their incubation with 100 U of DNase I (NEB) and 50 U of RNase A (Qiagen) at 37°C for 1 h. As controls for the reaction, DNA (500 ng of plasmid pAU-TM) and RNA (500 ng of IAC *in vitro* transcript, see below) were incubated under the same conditions. The ability of MS2 PLP to resist the action of nucleases was controlled using agarose gel electrophoresis (1%).

Prepared MS2 PLP were diluted with 10 mM Tris containing 100 mM NaCl and 1 mM EDTA supplemented with 0.05 mg/ml of bovine serum albumin (BSA; Life Technologies) to a final concentration of 1 × 10^6^/μl, verified by TEM, aliquoted (70 μl) and stored at -80°C.

### RT-qPCR for Detection and Quantification of MS2 PLP

A two-step, duplex, RT-qPCR was optimized for MS2 PLP and internal amplification control (IAC) detection and quantification. To distinguish false and truly negative results *in vitro* transcribed RNA (IAC) was included in the RT-qPCR assay. The IAC-specific sequence was derived from the genomic sequences of two plants – nepenthes (*Nepenthes ampullaria*, GenBank accession No. GQ338261.1), and potato (*Solanum tuberosum*, GenBank accession No. AF483209) and from *Mycobacterium avium* subsp. *paratuberculosis* (GenBank accession No. X70277). The DNA-containing IAC sequence and DNA-containing fragment encoding the specific control sequence were prepared according to Vojkovska et al. (2015). *In vitro* transcription of the IAC and the fragment encoding the specific control sequence derived from thylacine and the moa bird was performed as was previously described Vasickova et al. (2012). *In vitro* transcripts were stored at -80°C.

Reverse transcription was carried out using PrimeScript Reverse Transcriptase (Takara, Shiga, Japan) with slight modifications to the manufacturer’s protocol. The RT mixture (20 μl) contained 0.5 nmol of dNTP mix (Serva, Heidelberg, Germany), 20,000 molecules of IAC RNA, 2 pmol of both reverse primers (TM R and NP R; **Table 1**), 4 μl of PrimeScript reaction buffer, 5 U of reverse transcriptase, 1 U of RNase inhibitor (NEB) and 5 μl of isolated RNA. The reaction was performed at 50°C for 1 h followed by 75°C for 15 min and a cooling step at 10°C.

The optimized duplex qPCR assay was run in duplicate for each analyzed sample in a total volume of 20 μl. The reaction mix contained 10 μl of LightCycler 480 Probes Master (Roche), 5 pmol of the NP F and NP R primers, 10 pmol of the TM F and TM R primers, 4 pmol of the IAC probe and 1 pmol of the NP P probe, and 5 μl template cDNA. 1 U of Uracil DNA Glycosylase (Roche) was used in each qPCR reaction to avoid possible carry-over contamination. qPCR was performed using the Roche LightCycler 480 under the following reaction conditions: initial denaturation at 95°C for 10 min, followed by 45 cycles at 95°C for 10 s, 60°C for 30 s and 72°C for 10 s. The subsequent analysis of results (quantification) was carried out using the “Fit point analysis” option of the LightCycler 480 Software release 1.5.0 (version 1.5.0.39).

Quantification standards were prepared from a 10-fold dilution of *in vitro*-prepared RNA transcripts of the fragment encoding the specific control sequence derived from thylacine and the moa bird in the range of 5 × 10^9^ genome copies/5 μl to 5 × 10^1^ genome copies/5 μl. RNA *in vitro* transcripts were quantified by fluorimetry using the Qubit RNA BR Assay Kit (Life Technologies) and Qubit 3.0 fluorometer (Life Technologies) according to the manufacturer’s instructions.

MS2 PLP were lyzed in triplicate by heating at 95°C for 5 min (Cheng et al., 2006) and 5 μl of undiluted, ten-fold and hundred-fold diluted (RNase-free H_2_O, 40 U/ml RNase inhibitor, NEB; 50 ng/μl carrier RNA, Life Technologies) lyzed MS2 PLP were used as template RNA for the RT-qPCR reaction. RT was carried out in triplicate for each dilution and qPCR was done in triplicated for each sample.

### Artificial Contamination, Isolation, Detection, and Quantification of MS2 PLP in Different Matrices and Calculation of Analytical Efficiency

Swab, liver tissue, serum, feces, and vegetable samples were artificially contaminated with the 5 μl of MS2 PLP (5 × 10^6^ particles). RNA from liver tissue (50 mg aseptically taken from three different inner-liver locations) was isolated using the RNeasy Mini kit (Qiagen), while RNA from serum (200 μl) and feces (200 μl from 250 mg of feces resuspended in 2.25 ml of phosphate-buffered saline buffer – PBS) was isolated using the QIAamp Viral RNA kit (Qiagen) with slight modifications as described previously (Vasickova et al., 2011, 2012). RNA from leafy green vegetables (25 g) and swabs (100 cm^2^, cotton swab rinsed in 5 ml of PBS) was isolated using NucliSENS Magnetic extraction reagents (bioMérieux, Durham, NC, USA) according to ISO/TS 15216-1. Elution volumes of isolated RNA were 100 μl for swab and vegetable samples and 60 μl for liver tissue, serum and feces samples. RT-qPCR for detection of MS2 PLP was performed as described above.

Subsequently, 15 μl of MS2 PLP from aliquots which were used for artificial contamination of each sample were thermally lyzed (95°C for 5 min) and 5 μl of lyzed solution was directly added in duplicate to RT-qPCR. This approach allowed calculation of the exact *z*-value (see below). The extraction efficiency of each sample was calculated as follows:

$$\text{Extraction efficiency }(\%)\text{ = }\frac{\left(\frac{x}{5}\times y\right)}{z}\times 100$$

where *x* = number of MS2 PLP in the sample quantified in RT-qPCR, *y* = volume in which the isolated RNA was eluted and *z* = number of MS2 PLP added to the sample prior to nucleic acid isolation. To determine the quantity of isolated RNA molecules, RT-qPCR quantity value (*x*) is divided by 5 to obtain the quantity of RNA molecules in 1 μl (RT-qPCR uses 5 μl of template RNA), followed by multiplication by elution volume (*y*).

## Results

### Production of MS2 PLP

**FIGURE 1:**
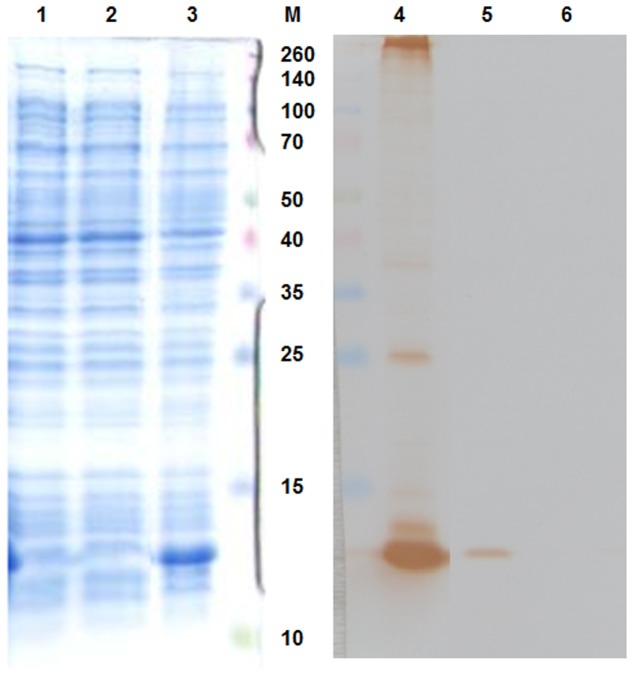
**Sodium dodecyl sulfate-polyacrylamide gel electrophoresis (SDS-PAGE – **left part** of the picture) and Western blot (**right part** of the picture) results.** 1,6, *E. coli* BL21 (DE3) negative control; 2,5, IPTG-non-induced culture; 3,4, IPTG-induced culture. 13 kDa MS2 coat protein was massively produced in the IPTG-induced culture. M, marker Spectra multicolor broad range protein ladder (Fermentas), the marker values are in kDa.

**FIGURE 2:**
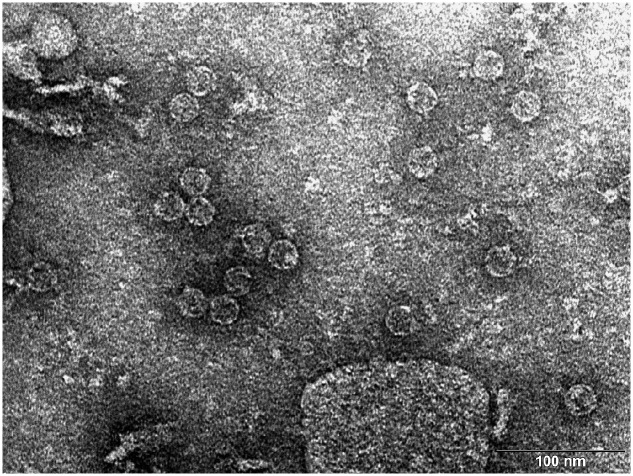
**Transmission electron microscopy (TEM) photograph of the intact MS2 phage-like particles (MS2 PLP) present in the supernatant after ultrasonic disruption of *E. coli* production cells.** MS2 PLP are about 27 nm in diameter. The scale is 100 nm.

The pAU-TM expression vector carrying all the necessary information for the production of MS2 PLP together with a unique control sequence was successfully prepared. The sequence of the expression vector was verified by sequencing (data not shown). The production of MS2 coat protein in cell culture after induction was controlled by SDS-PAGE and Western blot analysis (**Figure 1**). The results of these analyzes showed that the 13 kDa MS2 coat protein was produced in massive amounts in the IPTG-induced cell culture carrying the pAU2-TM expression vector. In contrast, only basal production of MS2 coat protein was observed in the control IPTG non-induced cell culture and no MS2 coat protein production was observed in the control *E. coli* BL21 (DE3) without the pAU-TM plasmid. The formation of intact MS2 PLP in the supernatant was inspected by TEM after ultrasonic disruption of collected *E. coli* cells (**Figure 2**). These results clearly showed spontaneous assembly of MS2 PLP in the induced cell culture.

### Purification of MS2 PLP

**FIGURE 3:**
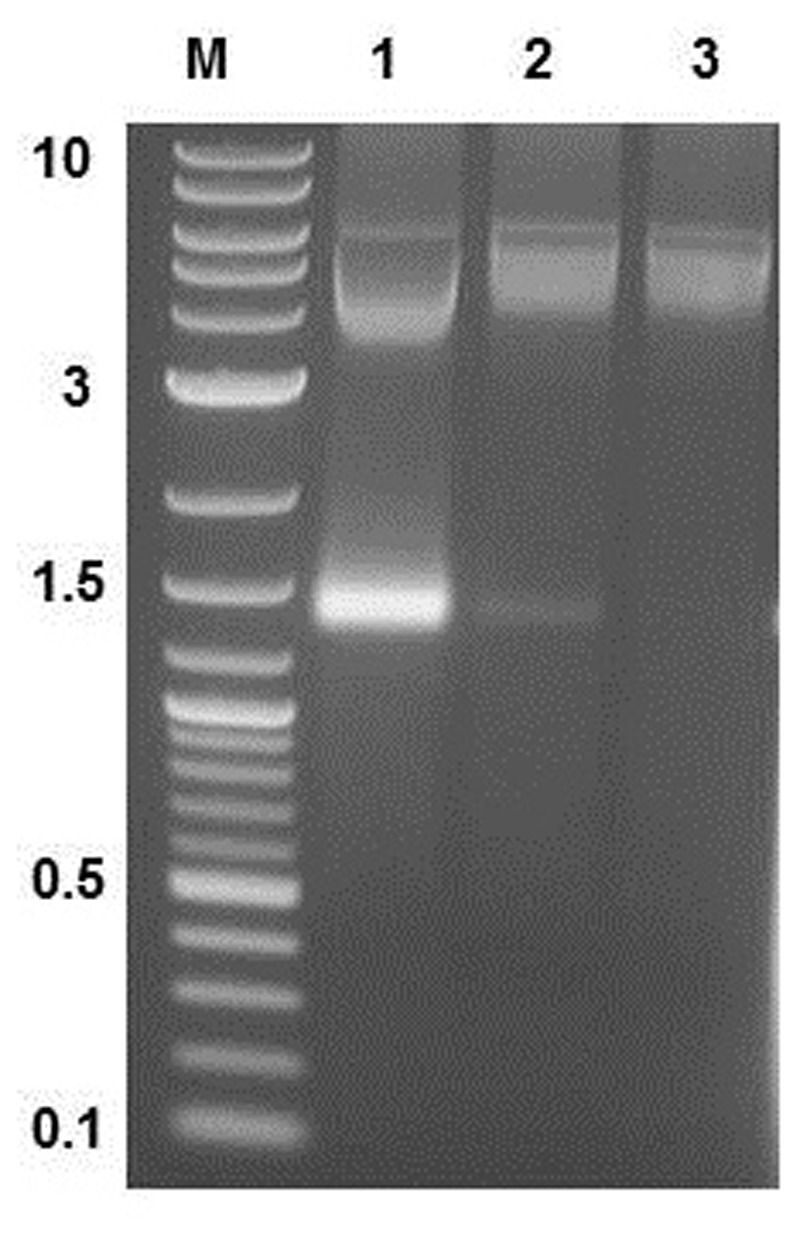
**Result of agarose gel electrophoresis (1%) detection of MS2 phage-like particles (MS2 PLP) in three layers removed after ultracentrifugation from the tube.** 1, top layer; 2, middle layer; 3, lower layer. M, marker 2-log DNA ladder (New England Biolabs, UK), the marker values are in kb. The top layer contained the highest amount of MS2 PLP (the band on the gel around 1.5 kb).

**FIGURE 4:**
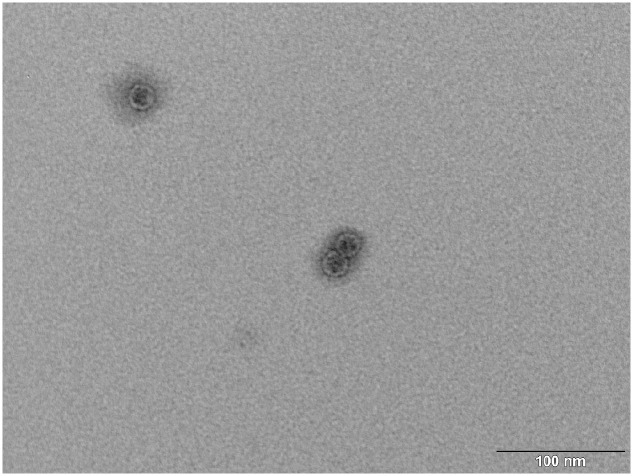
**Transmission electron microscopy (TEM) photograph of the intact MS2 phage-like particles (MS2 PLP) after purification steps.** The scale is 100 nm.

Three opalescent layers were distinguishable inside the ultracentrifugation tube after ultracentrifugation. All layers were removed and the presence of MS2 PLP in these layers was controlled by agarose gel electrophoresis (1%) and staining with ethidium bromide (**Figure 3**). It was found that the lower layer contained no MS2 PLP, the middle layer only a trace amount of MS2 PLP and that the top layer contained the highest amount of MS2 PLP. Collected MS2 PLP were further purified by dialysis. Dialyzed MS2 PLP was also controlled by agarose gel electrophoresis (1%) and staining with ethidium bromide. The band containing purified MS2 PLP was clearly visible (data not shown). The integrity of purified MS2 PLP was inspected by TEM (**Figure 4**).

### Quantification of MS2 PLP

**FIGURE 5:**
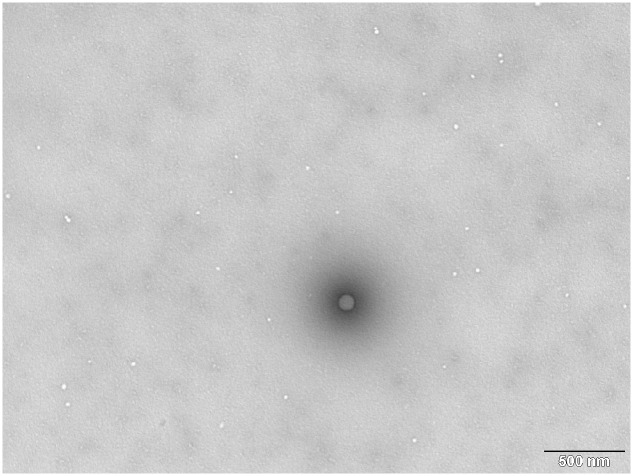
**Transmission electron microscopy photograph from TEM MS2 phage-like particles (MS2 PLP) quantification against latex standard.** The large black “dot” is the latex standard and the white dots are MS2 PLP. The scale is 500 nm.

The number of MS2 PLP was determined by UV spectrophotometry, TEM (**Figure 5**), fluorimetric measurement of protein concentration and RT-qPCR. The results of MS2 PLP quantification experiments are summarized in **Table 2**.

**Table 2:** The results of MS2 phage-like particles (MS2 PLP) quantification experiments.

Quantification method	UV spectrophotometry	TEM	Fluorimetry	RT-qPCR
Concentration of MS2 PLP/ml ± SD	2.5 × 10^13^ ± 2.24 × 10^11^	1.57 × 10^13^ ± not applicable	7.28 × 10^13^ ± 1.35 × 10^13^	2.89 × 10^12^ ± 2.39 × 10^11^


*TEM, transmission electron microscopy; RT-qPCR, reverse transcription polymerase chain reaction. SD, standard deviation.*

Values of MS2 PLP concentrations obtained by UV spectrophotometry, TEM and fluorimetry were comparable. In the case of RT-qPCR quantification of MS2 PLP, undiluted, 10-fold and 100-fold diluted suspensions of thermally lyzed MS2 PLP were tested in order to detect possible inhibitory effects of heat denatured proteins on RT-qPCR results. This dilution was done only in RT-qPCR quantification of MS2 PLP experiment in which high quantity of MS2 PLP was expected. Because described RT-qPCR system prefers to amplify control sequence before IAC sequence, in case that the quantity of MS PLP was very high no IAC was detected. When the sample was diluted, IAC could be detected and the results were therefore valid. In calculation of extraction efficiency of MS2 PLP from spiked matrices no dilution was done because the MS2 PLP quantity was not so high and the IAC was reliably detected even in undiluted samples. Amplification of IAC had to be successful otherwise the results were not valid.

RT-qPCR quantification method was chosen as the most accurate method because it was the only method capable of identifying a specific control RNA encapsulated inside the MS2 PLP.

### Verification of the Stability of MS2 PLP in the Presence of Nucleases

The stability of MS2 PLP was checked by agarose gel electrophoresis (1%) and staining with ethidium bromide (data not shown). These analyzes clearly showed that only the MS2 PLP were stable in the solution with high concentrations of DNase and RNase whereas naked DNA and RNA molecules were quickly degraded in this suspension.

**FIGURE 6:**
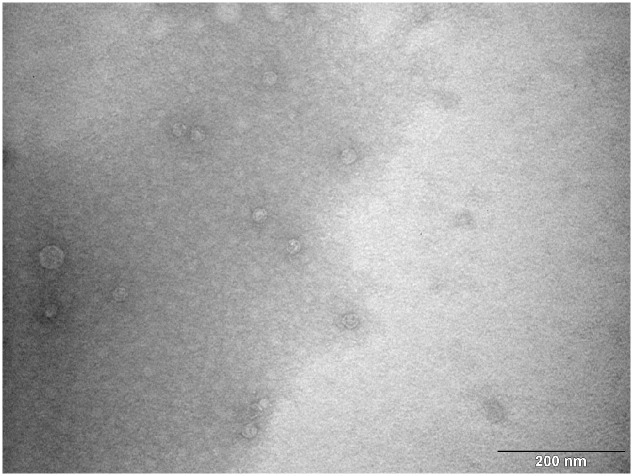
**Transmission electron microscopy photograph of homogenous and non-aggregated MS2 phage-like particles (MS2 PLP).** The scale is 200 nm.

According to results of RT-qPCR quantification prepared MS2 PLP were diluted to a final concentration of 1 × 10^6^/μl. The prepared solution was verified by TEM. TEM inspection revealed that prepared MS2 PLP did not form aggregates and that the suspension was homogeneous (**Figure 6**).

### Artificial Contamination and RT-qPCR Detection of MS2 PLP from Different Matrices

MS2 PLP were added in the amount of 5 × 10^6^ particles to the different types of matrices (swabs, liver tissue, serum, feces, and leafy green vegetables) to reveal their ability to serve as PCV in the RT-qPCR detection of enteric RNA viruses. According to results of RT-qPCR the extraction efficiencies were calculated for each individual matrix. The obtained efficiencies from swabs, liver tissue, serum, feces and leafy green vegetables were 41.60, 5.28, 77.04, 8.77, and 4.29%, respectively (**Table 3**).

**Table 3:** Extraction efficiency of MS2 phage-like particles (MS2 PLP) isolated from different types of matrices artificially contaminated with 5 × 10^6^ MS2 PLP per sample.

Matrix	Number of MS2 phage-like particles isolated from samples^1^		Positive samples/analyzed samples	Extraction efficiency	
			
	Mean	*SD*		Mean	*SD*
Swab	2,66 × 10^6^	7,74 × 10^5^	20/20	41,60%	12,09
Liver tissue	1,45 × 10^6^	7,72 × 10^5^	20/20	5,28%	2,63
Serum	6,10 × 10^6^	1,76 × 10^6^	20/20	77,04%	22,22
Feces	7,40 × 10^5^	1,80 × 10^5^	20/20	8,77%	2,14
Vegetable	2,99 × 10^5^	1,89 × 10^5^	18/18	4,29%	2,71


*^1^Total amount of MS2 PLP from each sample was determined according to the calibration curve included in each RT-qPCR experiment. RT-qPCR was run in duplicate for each sample. Nucleic acid isolation efficiency was calculated according to the formula described within the text. SD, standard deviation.*

## Discussion

RT-qPCR-based detection and quantification of RNA viruses for diagnostic purposes requires strict control to avoid inaccurate results. Bacteriophage MS2 and its derivatives (including MS2 PLP) are not the only possible useful PCV for the RT-qPCR detection of enteric RNA viruses. Many different viruses were used as PCV for the RT-qPCR detection of HAV and NoV from food matrices, e.g., Murine norovirus 1 (MNV-1) (Martin-Latil et al., 2012; Coudray et al., 2013; Hennechart-Collette et al., 2014), Feline calicivirus (FCV) (Mattison et al., 2009; Di Pasquale et al., 2010), Mengovirus, San Miguel sea lion virus serogroup 17 (SMSV-17) (DePaola et al., 2010) or echo type 9 virus (Nishida et al., 2007). According to ISO/TS 15216 recommendation, Mengovirus shall be used as a PCV in RT-qPCR determination of HAV and NoV in food matrices (ISO/TS 15216-1, 2013). However, recent studies have utilized MNV-1 instead of Mengovirus as the most appropriate virus for validation of HAV detection (Hennechart-Collette et al., 2015). Ideally, each genus of enteric RNA virus should have its own PCV that most closely matches its specific properties. However, such an approach is impossible in the case of routine detection of a broad spectrum of RNA viruses in different matrices. For example it was suggested that the most suitable PCV for NoV would be MNV-1, despite the recovery rates of MNV-1 were significantly different in bottled water and tap water (Hennechart-Collette et al., 2014). On the other hand, some recent studies have shown that MNV-1 virus mimicked the behavior of NoV during sample processing to the least extent and with the most dissimilar recovery to NoV (Gentry-Shields and Jaykus, 2015). Besides MNV-1, other PCV –Turnip crinkle virus (TCV), Mengovirus, Tulane virus and bacteriophage MS2 were also tested and bacteriophage MS2 and Tulane virus performed relatively well across all spiked levels (Gentry-Shields and Jaykus, 2015). Moreover, evaluation of two commercially available Mengovirus suspensions showed significantly different RNA extraction efficiency results. It was clear that differences in virus source (e.g., method of propagation) can drastically impact the efficiency of RNA extraction (Gentry-Shields and Jaykus, 2015). Also in this respect, MS2 PLP have an additional advantage, because they are produced strictly in *E. coli* BL21 (DE3) cells, which excludes the differences in virus source and provides for high quality, standardized PCV stocks.

MS2 PLP are appropriate candidates for PCV only in the case of detection of structurally similar RNA viruses – small, non-enveloped ssRNA viruses with icosahedral structure, among which the majority of common enteric viruses belong – to closely mimic their physiochemical properties during the nucleic acid extraction step. Therefore, MS2 PLP can be good PCV in the detection of HAV, HEV or NoV from food matrices. Although their production is not a trivial task, once they can be produced, a high amount is obtained. In comparison with commercially available PCV the price of self-producing MS2 PLP is significantly lower. Moreover, MS2 PLP can be stored for a long time without degradation (WalkerPeach et al., 1999; Beld et al., 2004; Hietala and Crossley, 2006; Wei Y.X. et al., 2008; Yu et al., 2008; Zhan et al., 2009; Song et al., 2011). Another positive feature of MS2 PLP is that they are not infectious and thus working with them is safe for laboratory personnel. Their advantage is the possibility to pack any ssRNA sequence into a capsid which can then serve as a specific control sequence.

On the other hand, a negative feature of MS2 PLP is that they do not form plaques because they do not carry a gene for lysis. Therefore, their number cannot be easily determined using a conventional plaque assay. For this reason, indirect methods for MS2 PLP quantification are used. The number of MS2 PLP may be determined using the Avogadro constant, extinction coefficient of 1 OD_260_ = 0.125 mg/ml of MS2 bacteriophage and the molecular weight of 3 × 10^6^ (DuBois et al., 1997; Cheng et al., 2006; Wei B.J. et al., 2008). This indirect method of enumeration of MS2 PLP was used together with flourimetry and with direct calculation of viral particles by TEM. The TEM quantification has not been used in any previous publications dealing with MS2 PLP. This was probably due to their relatively small size (around 27 nm) because quantitation of virus particles with TEM was used mostly for viruses larger than 40 nm (Stinski et al., 1979; Kwon et al., 2003; Wei et al., 2007; Weidmann et al., 2011). The concentrations of MS2 PLP obtained using UV spectrophotometry, fluorimetry and TEM were consistent.

Using an RT-qPCR method, the measured number of MS2 PLP was about 1 log_10_ lower (2.89 × 10^12^ MS2 PLP/ml) than by previously described methods. This indicates that the ratio of MS2 PLP carrying specific control RNA sequence in a population of prepared MS2 PLP is only around 10%. This result can be explained on the basis of the latest findings regarding the capsid assembling mechanism of bacteriophage MS2 which use a sophisticated coassembly process where the RNA genome interacts at multiple sites with the capsid proteins during assembly (Dykeman et al., 2013). Therefore, it is probable that the majority of the prepared MS2 PLP contain non-specific cellular RNA rather than specific control RNA, which is in concordance with previous findings (Pickett and Peabody, 1993). Based on these findings, the RT-qPCR method was selected for quantification of prepared MS2 PLP because it was the only method capable of identifying a specific control RNA encapsulated inside the particle.

Addition of a specific number of MS2 PLP – PCV – to each analyzed sample should provide information about the process of the sample analysis and therefore, it is possible to determine the extraction efficiency of RNA from each sample. Because false negative results may be caused not only by the failure of viral concentration steps and the nucleic acid extraction step but also by the inhibition of enzymatic reactions steps or failures in the elution, another control RNA molecule (IAC) was added to the RT-qPCR to detect possible inhibition on the level of RT-qPCR. Therefore, each isolated RNA sample should be first subjected to a RT-qPCR assay for detection and quantification of MS2 PLP with IAC. This first assay allows exact determination of the RNA extraction efficiency and also allows detection of potential inhibition in each analyzed sample. Then, additional RT-qPCR assays can be performed to detect various types of enteric RNA viruses. Based on this approach, it is possible to obtain reliable results for RT-qPCR detection of enteric RNA viruses.

To demonstrate the usefulness of MS2 PLP as PCV, MS2 PLP were added to different types of matrices, which commonly harbor viruses. The exact number of MS2 PLP added to samples (5 × 10^6^ particles) was chosen according to ISO/TS 15216-1 and ISO/TS 15216-2. Nucleic acid was isolated from different types of matrices using commercially available isolation kits. All isolation procedures were able to isolate viral RNA (PCV RNA) with an efficiency of higher than 1%, which was recognized as the threshold of successful isolation (ISO/TS 15216-1). When the extraction efficiency is lower than 1%, sample results are not valid and the sample must be retested. Although the purpose of this study was not to evaluate or to compare the performance of various RNA isolation procedures, some interesting findings were obtained. First, the extraction efficiency does not depend on the used isolation kit, as is obvious from the extraction efficiency of swab samples (41.60%) and vegetable samples (4.29%) both isolated with NucliSENS Magnetic extraction reagents (bioMérieux), or serum samples (77.04%) and fecal samples (8.77%), both isolated with QIAamp Viral RNA kit (Qiagen). The RNA extraction protocols are therefore well suited and the resulting extraction efficiency is much more dependent on concentration steps, which precede the isolation of viral RNA. For example, in the case of vegetable matrix, samples undergo a relatively complicated, time-consuming rinsing and concentration procedure prior to the nucleic acid isolation step, resulting in relatively low extraction efficiency. Identical isolation applied to swab samples without any pre-processing steps results in higher extraction efficiency of RNA (**Table 3**). Second, the composition of the analyzed sample has a crucial impact on extraction efficiency. The extraction efficiency from the serum matrix (77.04%), which is a relatively homogenous matrix, is almost 10-fold higher than extraction efficiency from feces (8.77%), which are not as homogeneous as serum samples and are expected to contain a higher concentration of inhibitory substances. A similar impact of sample composition is also obvious in the case of swab and vegetable samples (Abu Al-Soud and Radstrom, 2000; Radstrom et al., 2004; Schrader et al., 2012).

The use of PCV in RT-qPCR detection of RNA viruses in complex matrices is highly beneficial, since further increases the validity of the results. ISO/TS 15216 emphasizes the use of PCV (Mengovirus) in RT-qPCR detection of RNA viruses from food matrices (ISO/TS 15216-1, 2013; ISO/TS 15216-2, 2013). The use of appropriate PCV in RT-qPCR detection of RNA viruses from clinical samples is also necessary, but so far there is no ISO/TS. Therefore, the potential of MS2 PLPs as PCVs was tested in RT-qPCR detection of RNA viruses from food matrices together with clinical samples. Since there is still no universally accepted standard for clinical samples, some requirements (exact number of MS2 PLPs added to the samples, threshold of successful isolation) were adopted from standard applied in the analysis of food matrices (ISO/TS 15216-1). Moreover, MS2 PLPs also fulfill some additional requirements of ISO/TS 15216 – are non-enveloped, carry +ssRNA, have a similar size to the target viruses, are genetically distinct from the target viruses and their natural occurrence in tested sample is highly unlikely. On the other hand, MS2 PLPs do not meet the requirement of ISO/TS 15216-1 that PCV shall be a cultivable virus. This requirement is based on need to be able to maintain a sufficient supply of PCV in the laboratory by cultivating the PCV in cell culture and get more in-house prepared supplies of it. This can be a problem in laboratories where cultivation of viruses is restricted or in laboratories which do not have experience with the cultivation of viruses in cell cultures. In these cases, MS2 PLPs represents an alternative approach because they can be easily produces through bacterial cultures in high quantity. Moreover, this production approach of MS2 PLPs excludes the difference in virus source and provides for high quality, standardized PCV stocks.

This work took some generally accepted recommendations used in RT-qPCR detection of RNA viruses from food matrices and applied them also for clinical samples. This work does not represent a competitive approach for ISO/TS 15216. In case of food samples, using of MS2 PLPs in RT-qPCR detection exactly according to ISO/TS would require comparison of the PCV used in ISO/TS (Mengovirus) with the MS2 PLPs and also discussion regarding additional specific requirements for PCV with technical committee that developed the standard.

## Conclusion

The described RT-qPCR system including MS2 PLP represents a potent tool for the complex monitoring of sample analysis. The design of the system with the inclusion of control RNA and IAC sequences allows their usage in any diagnostic procedure for the molecular detection of non-enveloped RNA viruses from different types of matrices. The testing of the developed RT-qPCR system on different matrices of clinical and food origin artificially contaminated with MS2 PLP showed that it is robust enough to provide reliable and precise data.

## Author Contributions

Conception and design of the work: PM, PV, and PeK; Acquisition of data: PM, RT, HM, PK, and TV; Interpretation of data: PM, PV, and PeK; Drafting the work: PM; Revision of the manuscript: PV and PeK; All authors approved the version to be published in *Frontiers in Microbiology* and agreed to be accountable for all aspects of the work.

## Conflict of Interest Statement

The authors declare that the research was conducted in the absence of any commercial or financial relationships that could be construed as a potential conflict of interest.

## Footnotes

1 http://blast.ncbi.nlm.nih.gov/Blast.cgi

2 http://eu.idtdna.com/calc/analyzer
